# The Achilles Tendon Rupture in Basketball: Systematic Review of the Present Literature

**DOI:** 10.1055/s-0041-1733988

**Published:** 2021-09-14

**Authors:** Berta Alegre, Roberto Seijas, Pedro Alvarez, Albert Pérez

**Affiliations:** 1 Instituto Cugat Hospital Quiron Barcelona, Barcelona, Spain; 2 Medicine Department, Universitat Internacional de Catalunya, Barcelona, Spain; 3 Fundación Garcia Cugat, Barcelona, Spain; 4 Mutualitat Catalana de Futbolistas (FCF), Real Federación Española de Futbol, Madrid, Spain

**Keywords:** Achilles' tendon rupture, basketball, review, sport

## Abstract

**Background** The most common cause of the Achilles tendon ruptures in the U.S. population is sports, basketball is one among them. It is not one of the most frequent injuries of this sport, but it is one of the most serious and impactful in its practice.

**Purpose** The objective of this work is to gather evidence and evaluate the relationship between Achilles' tendon ruptures and basketball.

**Study Design** This study designed as a systematic review.

**Material and Methods** A search of literature in the databases PubMed, Cochrane Library, and ClinicalTrials.gov was conducted during January and February 2020.

**Results** After the search and selection, three relevant articles were obtained due to their epidemiological content, Raikin et al and Lemme et al. In 2013 and 2018, they, respectively, conducted studies in U.S. populations, establishing sport as the most frequent injury mechanism in Achilles' tendon ruptures. It was analyzed according to injury mechanism, gender, body mass index (BMI), and age. In both studies, the lesion resulted more frequently in men and the sport involved in most of the cases was basketball (32 and 42.6%, respectively). In 2019, Lemme et al published an epidemiological design analyzing the Achilles tendon ruptures in the National Basketball Association (NBA), identifying risk factors and results in professional players. After suffering the injury, 36.8% of the patients had to retire or were holders in less than 10 games to the rest of their sports career.

**Conclusions** The relationship between basketball and Achilles' tendon rupture is clear in the general population. Despite this, new studies are required to complement the evidence obtained so far.

Currently, according to data from the Federation of International Basketball Associations (FIBA), more than 450 million people in the world practice basketball, 1 thus ranking among the five most popular sports on the planet.

Basketball in Spain is the second most popular team sport as the most federated after football. There are 3,619 clubs with 385,100 licenses of which 36% correspond to women and 66% to men. 2 Spain ranks as the second-best national team in the FIBA World Ranking after the United States in men, and third after the United States and Australia in women. 1

The most frequent injuries in the practice of this sport are those of hands and shoulders in upper limbs, along with ankles and knees in lower limbs. The Achilles tendon rupture does not stand out for its frequency, but rather for its severity, making it impossible for 20.5% of those affected to return to the usual practice of basketball in the case of professionals. 3

Sports activity is the most frequent cause of Achilles tendon rupture. In the United States, basketball is the sport in which the injury in question most frequently occurs, while in Europe it is soccer. 3

It occurs more frequently in adults under 55 to 60 years of age and is related, in most cases, to basketball. This makes it responsible for 48% of the ruptures related to sports, and 32% of all ruptures according to Raikin et al. 4 It has also been related to gender, body mass index (BMI), ethnicity, and both the practice of professional and amateur sports. 4

The objective of this work is to assess the relationship between Achilles' tendon ruptures and basketball.

## Material and Methods

This systematic review was conducted according to the Preferred Reporting Items for Systematic Reviews and Meta-Analyses (PRISMA) guidelines using a PRISMA checklist. Neither protocols nor registration have been assessed.

During January and February of 2020, a literature search was conducted on the prevalence of Achilles tendon rupture in basketball. Multiple databases were used such as PubMed, Cochrane Library, and ClinicalTrials.gov. The electronic search strategy used was “Achilles' tendon rupture and basketball.” Studies were screened by title and/or abstract to determine study eligibility based on inclusion criteria. The inclusion criteria were descriptive epidemiology studies that described prevalence of Achilles' tendon rupture in professional or amateur basketball players.

We have considered the following as inclusion criteria: all those published articles that contain the keywords, with data that highlight the relationship between the Achilles rupture and basketball. Exclusion criteria include systematic review articles, case reports, series where sport was not listed and where basketball was not included, series that did not present epidemiological data, and articles published in languages other than English or Spanish.

After the search, 24 results were found that were filtered according to the year of publication. The literature review shows an increase in publications from the year 2013 compared with the limited publication of studies in previous years. It was then decided to apply, saying year as a temporary filter obtaining 13 articles. Subsequently, the resulting articles were evaluated by title and abstract without considering the type of study, outcomes, gender, and/or interventions performed. Subsequently, 10 articles discussing other topics, such as treatment or prognosis of the injury, were excluded.

Finally, the following three articles were selected for their epidemiological content of relevance. First, “Epidemiology and Video Analysis of Achilles Tendon Ruptures in the National Basketball Association” of Lemme et al 3 published in August 2019 by *The American Journal of Sports Medicine* . Second, “Epidemiology of Achilles Tendon Ruptures in the United States” of Lemme et al 5 published in November 2018 by *Orthopedic Journal of Sports Medicine* . And finally, “Achilles Tendon Injuries in a United States Population” by Raikin et al 4 in April 2013 in *Foot & Ankle International magazine* . The three above are descriptive epidemiology studies with good quality assessment by the National Institute of Health (NIH) quality assessment tool ( Table 1 ).

**Table 1:** National Institute of Health quality assessment tool ratings

Authors (year)	Quality rating
Lemme et al 3 (2019)	Good
Lemme et al 5 (2018)	Good
Raikin et al 4 (2013)	Good

**Fig. 1:**
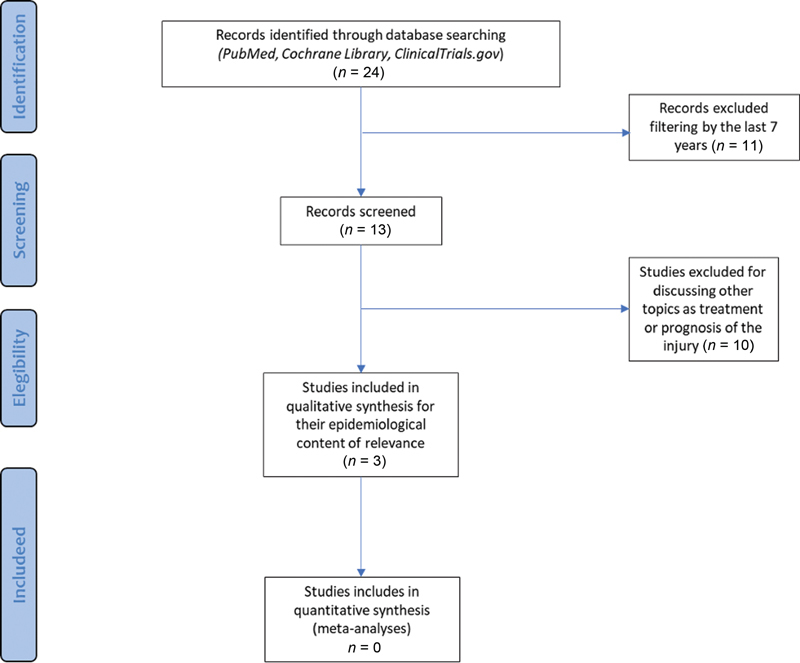
PRISMA (Preferred Reporting Items for Systematic Reviews and Meta-Analyses) flow diagram.

The search and selection are detailed in Fig. 1 . Independent data collection process was conducted.

## Results

After the search and selection, three articles were obtained, all of the epidemiologically descriptive designs 3 4 5 are presented in Table 2 with a summary of the most relevant aspects. Only the study by Lemme et al, published in 2019, specifically deals with the Achilles tendon rupture in basketball, specifically in the National Basketball Association (NBA) of the United States.

**Table 2:** Epidemiological designs

Authors (year)	Participants	Source	Incidence	Total cases
Lemme et al 3 (2019)	National Basketball Association players between the 1969 to 1970 and 2017 to 2018 seasons	Public data obtained from injury records, press communication, and player profiles	0.92 per 450 people-year a	44 cases between the 1969 to 1970 and 2017 to 2018 seasons
Lemme et al 5 (2018)	1.6 billion people-year at risk between 2012 and 2018	National Electronic Injury Surveillance System (NEISS)	2.1 per 100,000 people-year	32,906 cases between 2012 and 2016 42.6% of the total related to basketball
Raikin et al 4 (2013)	Patients who visit an orthopaedic clinic in need of tertiary attention with diagnostic and surgical treatment of a torn Achilles' tendon between August of 2000 and December of 2010	International Classification of Diseases ninth edition code for the Achilles tendon rupture in Misys Healthcare Systems software	2.66 per 1,000 people-year b	406 cases between August 2000 and December 2010 32% of the total related to basketball

a Calculated in a population at risk of 450 players/sports season.

b According to data from Moller et al in 2001 6 included in the study.

The 2018 study by Lemme et al 5 and the 2013 study by Raikin et al 4 were included because of their epidemiological relevance and relationship to sport, specifically basketball.

The study by Lemme et al 5 determined the incidence and risk factors of the Achilles tendon ruptures in the United States. Patients registered in the National Electronic Injury Surveillance System (NEISS) database, who came to the emergency department with this lesion between 2012 and 2016, were selected. The incidence was calculated according to the following variables: gender, age, race, and analyzing other variables such as the location where the injury occurred. Likewise, cases were classified according to the mechanism of injury, performing a subanalysis of injuries with sport. A significant increase in incidence was detected from 2012 with 1.8 cases per 100,000 person-years to 2016 with 2.5 cases per 100,000 person-years ( *p* < 0.01), and a global incidence of 2.1 per 100,000 person-years. Of the 32,906 cases, 77.1% affected were men while the remaining 22.9% affected were women. The greatest increase in cases occurred in female patients between 40 and 59 years of age (78% of the increase). The most common injury mechanism detected was participation in sports or leisure activities (81.9%), with basketball being the most frequent cause among all (42.6%), followed by American football (9.9%), tennis (6.9%), and athletics/climbing/stretching (5.8%).

Raikin et al 4 reviewed 406 cases of the Achilles tendon rupture in the U.S. population. Patients who attended a tertiary care orthopaedic clinic with diagnosis and surgical treatment of the Achilles tendon rupture, between August 2000 and December 2010, and registered in the International Classification of Diseases 9th edition code for Achilles' tendon rupture in Misys Healthcare Systems software were selected. Patients >55 years of age and with BMI > 30 kg/m ^2^ were more likely to rupture the Achilles tendon in nonsport activity and more likely to have an initial misdiagnosis. Likewise, it was established that age and BMI were directly related to the time of diagnosis of the lesion. Of the 406 cases, 83% affected were men while 17% were women. Sports activity was responsible for 68% of injuries, in patients <55 years of age with 77% of injuries compared with 42% in patients >55 years of age. Basketball was the most involved sport, being responsible for 48% of the breaks related to the sport, and 32% of the total ruptures of the Achilles tendon. This is followed by tennis (13 and 9%) and football (12 and 8%). Other sports such as squash, volleyball, or soccer accounted for 31% of total injuries and 46.5% of those related to sports.

**Fig. 2:**
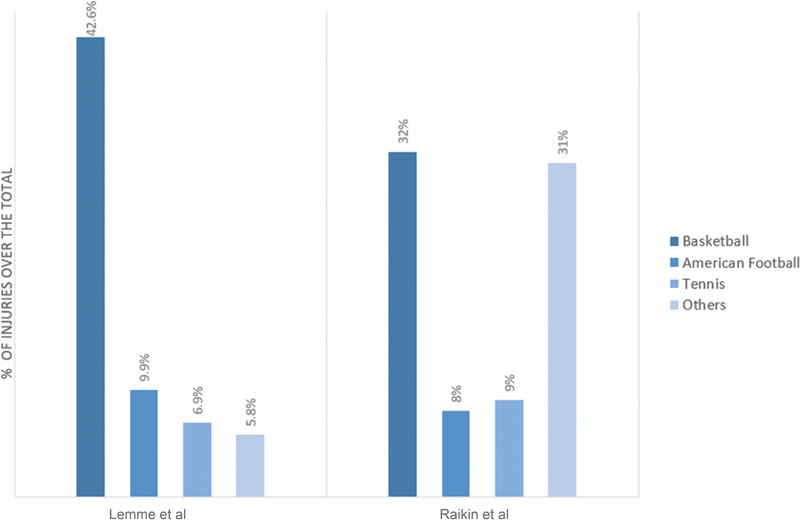
Percentage of sports injuries according to the sport performed on the total Achilles tendon tears.

The distribution by sport of the above-mentioned studies 4 5 is detailed in Fig. 2 .

In August 2019, Lemme et al 3 published a study based on epidemiology and video analysis of the Achilles tendon ruptures in the NBA, the American men's professional basketball league. The objective of this descriptive design was to identify risk factors for the Achilles' rupture in professional players of the best basketball league in the world, identifying cases from available public data on injury reports, press releases, and player profiles. Video analysis was used to identify the mechanisms of injury and the most frequent game situations where such injuries occurred. In 48 years, 44 players had Achilles' tendon ruptures. The average age of the injured players was 28.3 years with an average of 6.8 seasons played in the league. A higher prevalence was seen at the beginning of the season (27.3%), followed by the preseason (18.2%) and the end of the season (18.2%). The average recovery time was 10.5 months. In more than one-third of the cases (36.8%), the patients had to retire or were holders in less than 10 games to the rest of their sports career. The video analysis available in 12 cases showed that the injuries occurred without contact. The most frequent mechanism of injury was the beginning of the action starting from the resting position, with dorsiflexion of the foot, early knee flexion, and hip extension. In addition to age and time of the season, other characteristics of the lesion present in Table 3 were analyzed.

**Table 3:** Characteristics of the lesions analyzed of the 44 National Basketball Association players injured by Lemme et al in 2019 3

Variable	Category	Percentage of injury ( *n* )
Laterality of the injury	Right	50 (21)
	Left	50 (21)
Age (y)	<30	68.2 (30)
	>30	31.8 (14)
BMI	<25	40.9 (18)
	>25	59.1 (26)
Field position a	1–3 b	31.8 (14)
	4–5 b	68.2 (30)
Place of injury	Game	78.3 (29)
	Practice	21.7 (8)
Point in the season	Out of season	15.9 (7)
	Preseason	18.2 (8)
	Start of season	27.3 (12)
	Midseason	15.9 (7)
	End of season	18.2 (8)
	Postseason	4.5 (2)
Seasons played after recovery	0	20.5 (9)
	1 season	15.9 (7)
	≥2 seasons	63.6 (28)

Abbreviation: BMI, body mass index.

a Identified in 37 cases (84.1%).

b 1, point guard; 2, shooting guard; 3, small forward; 4, power forward; 5, center.

## Discussion

This review investigated the relationship between Achilles' tendon rupture and basketball. Numerous works coincide in the increase in Achilles' tendon ruptures in recent decades, assuming an incidence between 8.3 and 24 per 100,000 person-years 7 8 9 10 11 12 13 14 in the general population. It is necessary to highlight that the samples of the studies analyzed in this review were made in the U.S. population, with the limitation that it implies in terms of external validity. However, it should be noted that the professional basketball league in the United States has the best players in this sport, in addition to having a great social impact and a high demand in terms of sports load.

Gender was decisive in the analysis in addition to BMI and age. The present data show a greater number of injuries in men compared with women in the U.S. population as shown in Table 4 . Moreover, the only study of such injury in basketball professionals was performed on men, while there is not enough available data in women. In the future, the relationship of these injuries could be analyzed in the professional women's league of the Women's National Basketball Association (WNBA) in the United States.

**Table 4:** Gender distribution of Achilles' tendon tears

Authors (year)	Gender	Percentage of Injury ( *n* )
Raikin et al 4 (2013)	Masculine	83 (331)
	Feminine	17 (69)
Lemme et al 5 (2018)	Masculine	77.1 (25,374)
	Feminine	22.9 (7,533)

According to the data shown in the studies, sport is the most frequent injury mechanism, assuming 68 to 81.9% of injuries mainly in <55 years of age and BMI <30 kg/m ^2^ . 4 5 Basketball, causing 42.6% of injuries according to Lemme et al 5 and 32% of the lesions according to Raikin et al, 4 is considered as the sport in which the Achilles tendon ruptures most frequently occur in the United States. In contrast, soccer is the sport most involved in these types of injuries in Europe. 8 10 11

According to Fig. 2 , at the population level, basketball is the sport where the Achilles tendon breaks most frequently, followed by American football according to Lemme et al 5 and basketball and tennis according to Raikin et al. On the contrary, in professional practice, there are discrepancies. According to Parekh et al in the National Football League (NFL), there were 31 cases of the Achilles tendon rupture in the league over a period of 5 years (1997–2002). 15 This data are unquestionably superior to that of the NBA, where in 48 years, there were only 44 cases. Therefore, it is essential to differentiate Achilles' tendon ruptures in amateur players compared with professionals in basketball and other sports.

In the NBA in 48 years, there have been only 44 cases of the Achilles tendon ruptures. The average age of the injured is 28.3 years with a higher prevalence of injuries in players with BMI > 25 kg/m ^2^ . Precisely, the age between 28 and 32 years is when players assume their highest level of sports performance. Injuries occur mostly at the beginning of the season (27.3%) and in matches (78.3%). It is a more frequent injury in players of the power forward and BMI (body mass index) center positions (68.2%), and according to Amin et al, 16 with an average height of 200 cm (between 180.0 and 221.0).

In case of return to the sport, the average recovery time is 10.5 months. However, in 36.8% of the cases, the players retire or are holders in less than 10 games to the rest of their sports careers. In 20.5% of the cases, the players are forced to withdraw when they suffer this injury. Also, 15.9% will play only one more season after their recovery, and the rest will play two or more seasons after recovery (63.6%). Therefore, in professional basketball, the Achilles tendon rupture is not frequent but is an extremely serious injury.

Despite the above data, although there is sufficient evidence of the relationship between basketball and Achilles' tendon rupture, we are surprised by the low number of articles published globally, in relation to the number of cases that occur worldwide. Even establishing a clear relationship between basketball and injury, more studies are needed in both amateur and professional basketball players and in both genders.

## Conclusion

The relationship between Achilles' tendon rupture and basketball is clear. While among sports, at the population level, the most frequent injury mechanism is basketball, and at the professional level, there are uncertain results. Therefore, there is not enough evidence to determine the relationship between this sport and the injury in question, and further studies (both professional and amateur and in both genders) are needed to determine this.
